# A rare familial rearrangement of chromosomes 9 and 15 associated with intellectual disability: a clinical and molecular study

**DOI:** 10.1186/s13039-021-00565-y

**Published:** 2021-10-04

**Authors:** Natalya A. Lemskaya, Svetlana A. Romanenko, Mariia A. Rezakova, Elena A. Filimonova, Dmitry Yu. Prokopov, Alexander A. Dolskiy, Polina L. Perelman, Yulia V. Maksimova, Asia R. Shorina, Dmitry V. Yudkin

**Affiliations:** 1 Institute of Molecular and Cellular Biology SB RAS, Novosibirsk, Russia 630090; 2 State Scientific-Research Institute of Physiology and Basic Medicine, Novosibirsk, Russia 630090; 3 Federal State Budgetary Research Center of Virology and Biotechnology “VECTOR”, Federal Service for Surveillance on Consumer Rights Protection and Human Well-being (FBRI SRC VB “VECTOR”, Rospotrebnadzor), Novosibirsk Region, Koltsovo, Russia 630559; 4 Novosibirsk State Medical University, Novosibirsk, Russia 630090; 5 Novosibirsk Clinical City Hospital No. 1, Novosibirsk, Russia 630090

**Keywords:** Microdissection, Balanced reciprocal translocation, Trisomy, Next-generation sequencing, Prader–Willi/Angelman critical region, Resting-state functional MRI (fMRI), Undescended testis

## Abstract

**Background:**

There are many reports on rearrangements occurring separately in the regions of chromosomes 9p and 15q affected in the case under study. 15q duplication syndrome is caused by the presence of at least one extra maternally derived copy of the Prader–Willi/Angelman critical region. Trisomy 9p is the fourth most frequent chromosome anomaly with a clinically recognizable syndrome often accompanied by intellectual disability. Here we report a new case of a patient with maternally derived unique complex sSMC resulting in partial trisomy of both chromosomes 9 and 15 associated with intellectual disability.

**Case presentation:**

We characterise a supernumerary derivative chromosome 15: 47,XY,+der(15)t(9;15)(p21.2;q13.2), likely resulting from 3:1 malsegregation during maternal gametogenesis. Chromosomal analysis showed that a phenotypically normal mother is a carrier of balanced translocation t(9;15)(p21.1;q13.2). Her 7-year-old son showed signs of intellectual disability and a number of physical abnormalities including bilateral cryptorchidism and congenital megaureter. The child’s magnetic resonance imaging showed changes in brain volume and in structural and functional connectivity revealing phenotypic changes caused by the presence of the extra chromosome material, whereas the mother’s brain MRI was normal. Sequence analyses of the microdissected der(15) chromosome detected two breakpoint regions: HSA9:25,928,021-26,157,441 (9p21.2 band) and HSA15:30,552,104-30,765,905 (15q13.2 band). The breakpoint region on chromosome HSA9 is poor in genetic features with several areas of high homology with the breakpoint region on chromosome 15. The breakpoint region on HSA15 is located in the area of a large segmental duplication.

**Conclusions:**

We discuss the case of these phenotypic and brain MRI features in light of reported signatures for 9p partial trisomy and 15 duplication syndromes and analyze how the genomic characteristics of the found breakpoint regions have contributed to the origin of the derivative chromosome. We recommend MRI for all patients with a developmental delay, especially in cases with identified rearrangements, to accumulate more information on brain phenotypes related to chromosomal syndromes.

**Supplementary Information:**

The online version contains supplementary material available at 10.1186/s13039-021-00565-y.

## Background

Chromosomal abnormalities are a major driver of intellectual disability and multiple congenital anomalies. The abnormal chromosomes that cannot be identified are defined as marker chromosomes. A subset of rare small supernumerary marker chromosomes (sSMCs) consists of the material from two or three chromosomes as a result of meiotic malsegregation in carriers of a balanced reciprocal or Robertsonian translocation [1].

Karyotyping and microarray technology are primary tools in the genetic diagnosis of patients with a developmental delay. The resolution of G-banding is roughly 5–10 million base pairs (Mbp) [2]. Although conventional karyotyping can certainly identify the presence of large balanced chromosomal aberrations, origin identification for small supernumerary elements is beyond its resolution. The use of whole-chromosome painting probes in fluorescence in situ hybridization (FISH) allows to identify the source of euchromatin in a marker but does not determine boundaries of the rearrangement. Microarray technologies fail to identify balanced rearrangements and do not determine an exact breakpoint region. Breakpoints of chromosomal rearrangements are important for identifying and revealing an underlying gene function, an understanding of the mechanisms leading to chromosome rearrangements, and finding common features for breakpoint regions.

Here we applied a target approach to chromosome rearrangements by isolating chromosomes of interest by microdissection followed by next-generation sequencing (NGS) [3]. The analysis of such sequences allows to identify both the composition and breakpoint regions of aberrant chromosomes.

Here we describe a boy with a developmental delay and a complex sSMC arising from a 3:1 segregation error of a maternally derived translocation between chromosome 15q13.2 and chromosome 9p21.2, which led to trisomy of chromosome 15pter-q13.2 and 9pter-9p21.2. We conducted brain magnetic resonance imaging (MRI) to reveal anomalies caused by the chromosomal rearrangement. MRI is a critical tool for obtaining a description of changes in brain structure and for identifying the types of brain anomalies that are found in almost 60% of developmental-delay cases [4]. We described the proband’s pathology by analyzing his medical history, brain features revealed by the MRI, and the structure of the rearrangement. Genotype and phenotype correlations for the found karyotype pathology are discussed, specifically for the presence of supernumerary chromosome der(15)t(9;15)(p21.2;q13.2) for the first time. For the analysis, we used conventional and molecular cytogenetic methods, microdissection, and NGS to find the origin of the supernumerary element, to establish boundaries of the rearrangement, and to identify areas of partial trisomy.

## Materials and methods

### Chromosome preparation and cytogenetic analysis

Samples of peripheral venous blood were collected from the patients. The culturing of B -lymphocytes, metaphase chromosome preparation, and GTG-banding were carried out as described previously according to standard procedures and by standard trypsin/Giemsa treatment [5, 6]. FISH with human whole-chromosome sorted [7, 8] and microdissected probes was carried out as described previously [9–11]. Metaphase spreads were analyzed under an Olympus BX 53 microscope using the VideoTest Karyo 3.1 and VideoTest FISH 2.0 (iMicroTec, Russia) software. Karyotyping was performed by analysing 12–15 metaphases for every family member.

### Chromosome microdissection and amplification

Marker chromosomes were dissected as described earlier [12] by means of an Olympus IX 51 microscope and a micromanipulator, Eppendorf Transferman NK2. DNA of the microdissected chromosomes was amplified with the GenomePlex Complete Whole Genome Amplification Kit (Sigma-Aldrich, USA). Each microdissected library here was obtained from a single copy of an abnormal chromosome.

### DNA sequencing

The DNA libraries were prepared with the NEBNext Ultra II DNA Library Prep Kit for Illumina (Illumina, USA) and sequenced on the MiSeq (Illumina) platform (300 bp paired-end reads).

### Target region identification

The chromosome sequences were aligned to a reference genome, and target regions were identified with the DOPseq analyzer pipeline, which has been described earlier [13]. In brief, sequences of Illumina adapters and whole-genome amplification primers were removed from the reads obtained by the sequencing of microdissected chromosomes, and the sequences were filtered by length ≥ 20 in cutadapt 1.18 [14]. Then, pairs of reads were aligned to the latest build of human reference genome assembly GRCh38 using BWA-MEM 0.7.15 [15]. The aligned reads were filtered by mapping quality (MAPQ ≥ 20) and by alignment length (≥ 20 bp), and were merged into positions by means of BEDTools 2.26.0 [16]. Target regions were identified based on differences in the average distance between positions using the DNAcopy package [17].

### MRI and imaging data analysis

#### MRI procedures

The participants had brain MRI performed on a GE Discovery 750w (3T) System. The MRI analysis was conducted under general anesthesia (intravenous injection of 1% propofol) and included (a) a routine protocol (T2-WI, FLAIR); (b) high-resolution T1-WI (3D SPGR, sagittal plane): TR, 9.5 ms; TE, 4.2 ms; field of view (FOV), 256 mm; matrix, 256 × 256; slice thickness, 1 mm; and voxel size, 1 × 1 × 1 mm; (c) diffusion tensor MRI (diffusion tensor imaging [DTI]: axial EPI, 64 diffusion directions, 7 b0, b = 1000; FOV 25.6 cm, matrix 128 × 128, slice thickness 2.0 mm, 66–72 slices, TR 12 s, TE 110 ms; scan time 20 min); (d) resting-state fMRI (EPI-BOLD, axial plane, FOV 24 cm, matrix 80 × 80, slice thickness 3.0 mm, 42–48 slices, TR 2.5 s, TE 28–30 ms, flip angle 81, 200 volumes, 10 dummy scans, eyes closed, scan time 10 min). The total scan time was ~45 min.

#### Postprocessing of structural images (T1-WI), DTI, and fMRI

Automatic basic segmentation of structural T1 images and DTI postprocessing was performed using the FreeSurfer v6.0 and FreeSurfer TRAKULA v6.0 software (https://surfer.nmr.mgh.harvard.edu). Intracranial volume, total brain volume, volumes of white and gray matter, and volumes of individual subcortical structures were evaluated. The obtained volumes were compared with the standard values [18]. DTI postprocessing included motion correction, EPI distortion correction, coregistration with T1–WI in MNI (Montreal Neurological Institute), calculation of FA maps, calculation of mean FA values from individual regions of interest (ROIs; according to basic segmentation data), and, finally, tractography of 18 standard neuronal tracts.

Postprocessing of resting-state fMRI data was performed using the CONN v17 package (http://www.conn-toolbox.org). fMRI preprocessing included the removal of the first 10 volumes, slice-timing correction, smoothing, EPI distortion correction, coregistration with T1-WI in MNI at 3 × 3 × 3 mm final resolution. Next, ROI-by-voxel connectivity analysis was performed. Default mode network (DMN) nodes served as ROIs on the basis of existing data (Additional file 1: Table S1).

## Results

### The clinical report

A phenotypically normal couple had a child with signs of intellectual disability and a number of physical abnormalities. The boy was born of the first pregnancy at 39 weeks that proceeded without any pathology. The weight at birth was within the reference range (2880 g). The Apgar score of the newborn assessment test was 8–9 out of 10. At birth, a congenital defect of the urinary system was revealed: a right megaureter. Additionally, at age 6, bilateral cryptorchidism (undescended testes) was noted. By this age, secondary chronic pyelonephritis was described in the stage of clinical and laboratory remission after surgical treatment of obstructive megaureter. The child had delayed motor development (sitting up at 8 months and starting to walk after 2 years) and delayed speech development (saying first words at 4 years, and not speaking in sentences at 7 years). At age 6, the patient underwent electroencephalography, which revealed mild diffuse changes in bioelectrical activity; interhemispheric asymmetry and epileptic activity were not detected. At 7 years, the proband had normal physical development (between 25th and 75th percentiles) and the asthenic body type with a normal body ratio. During a physical examination for this study, the 7-year-old patient showed stigmergy: the skull had a hydrocephalic shape with a high forehead, hypertelorism, convergent strabismus, large low-set protruding ears, peg-shaped teeth, macrostomia, and diastema. The patient had a shaky gait with signs of ataxia.

The patient showed periods of a fixated look, had unstable attention, and was easily distracted. The child had difficulty communicating and interacting with other people and did not follow directions. The patient was found to be emotionally labile with severe motor hyperactivity, which is expressed in restlessness and reduced attention.

### Cytogenetic analysis

**Fig. 1:**
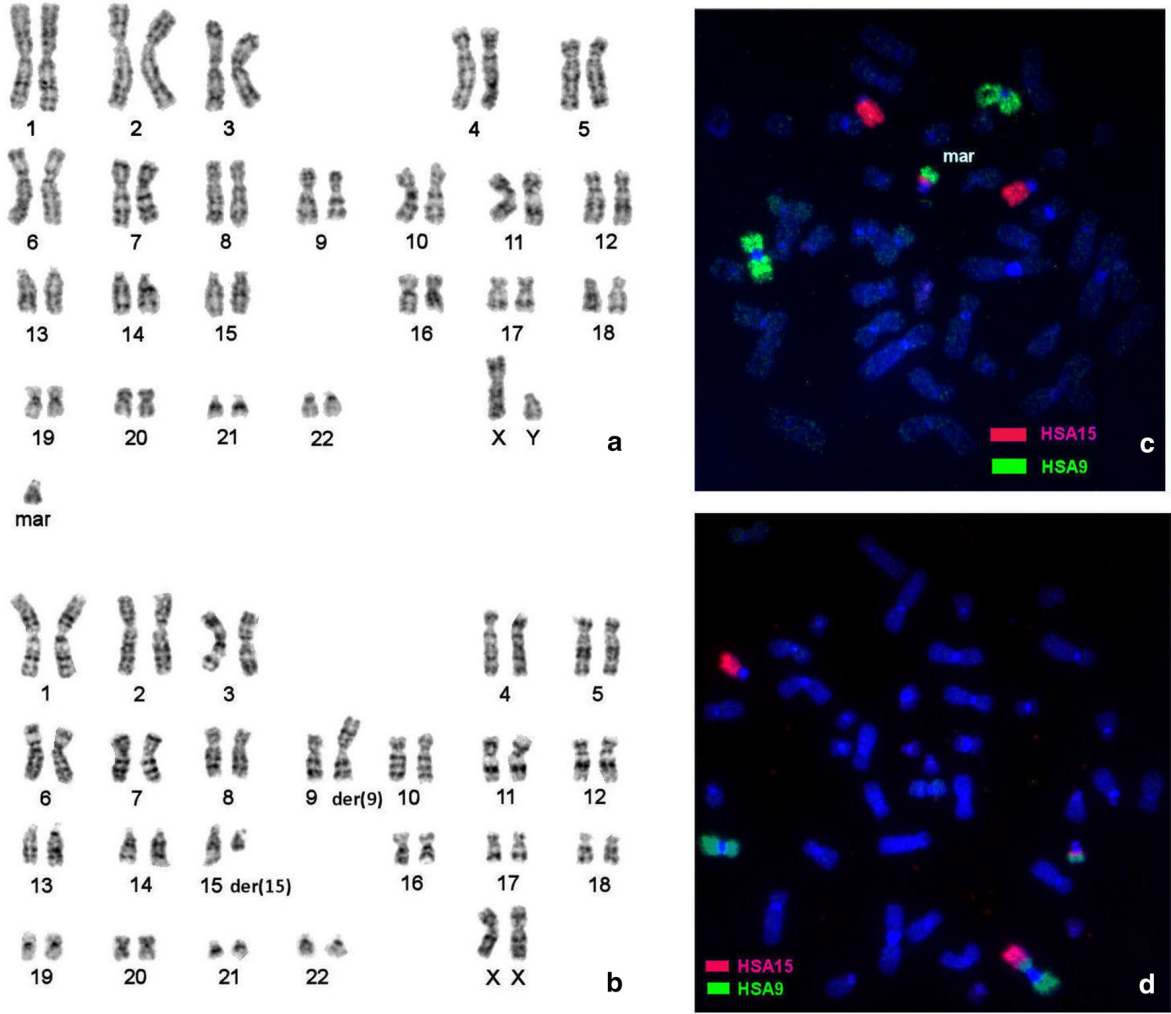
GTG-banded karyotype of **a** the proband and **b** his mother; FISH with painting probes of human chromosomes 15 (red signal) and 9 (green signal) on metaphase chromosomes of **c** the proband and **d** his mother

A standard cytogenetic analysis of the proband revealed the presence of a supernumerary marker chromosome in 100% metaphases without mosaicism (Fig. 1a).

An analysis of the father’s karyotype revealed no chromosomal abnormalities, while it was found that the mother had 2 abnormal chromosomes in all metaphases (Fig. 1b). The supernumerary marker of the proband was characterized and found to be a derivative of the mother’s chromosome 15. FISH with chromosome-specific probes showed that the marker consists of fragments of chromosomes 9 and 15 ( Fig. 1c), while the mother is a carrier of balanced reciprocal chromosomal translocation t(9,15)(p2;q1) (Fig. 1d).

### Single-chromosome sequencing of microdissected chromosomes

Microdissected-chromosome libraries were prepared for the derivative chromosome of the patient [homologous to his mother’s der(15)] and for the large derivative chromosome 9, der(9), of the mother. The accuracy of the microdissection was verified in experiments on reciprocal FISH of the obtained probes (data not shown).

**Fig. 2:**
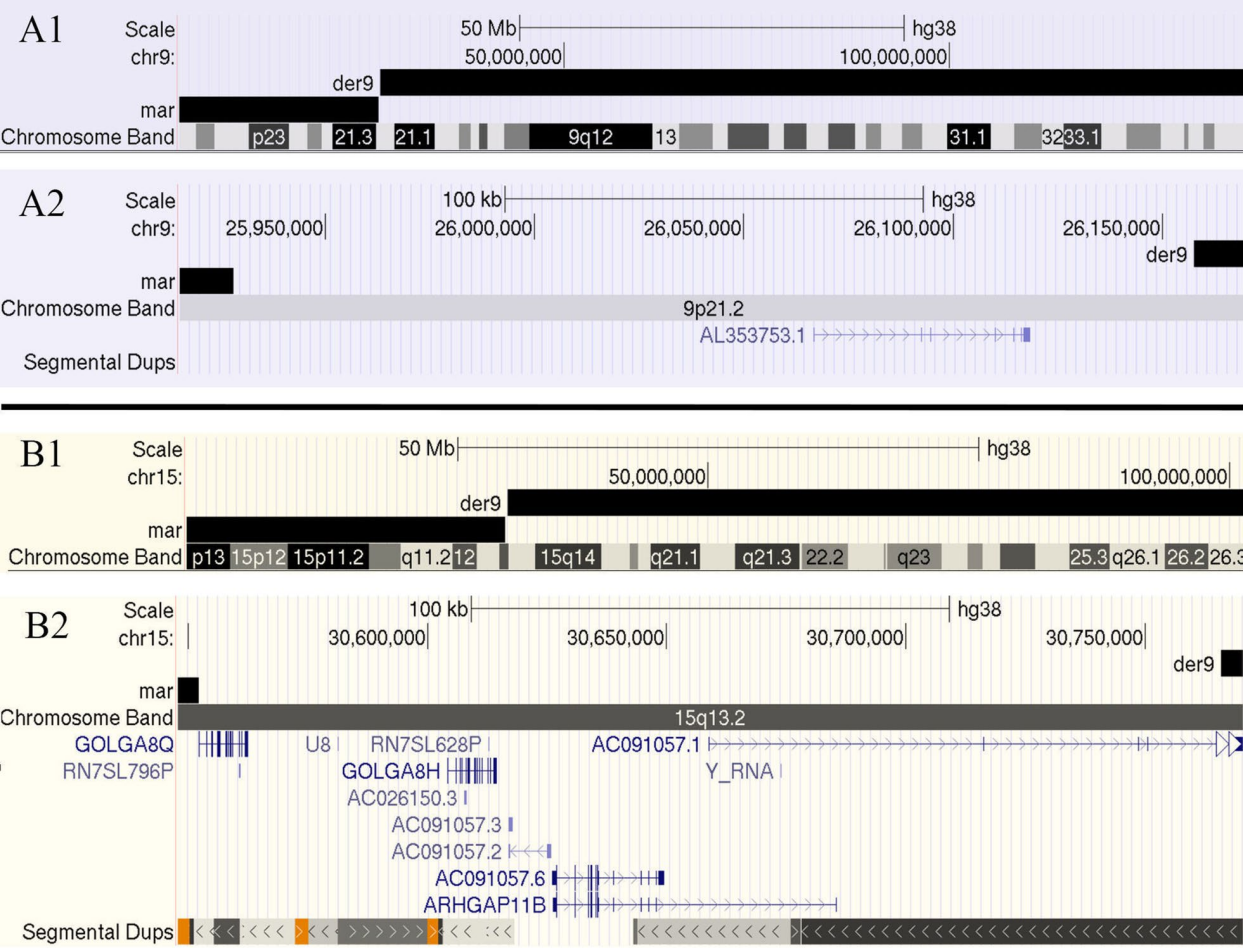
UCSC Genome Browser (https://genome.ucsc.edu/) representation of the breakpoint region on HSA9 (**A1**) and its enlarged fragment (**A2**) and on HSA15 (**B1**) and its enlarged fragment (**B2**). der9: the mother’s chromosome, mar: the proband’s derivative chromosome

A total of 50,206 (25,203,412 bp) and 48,699 (24,446,898 bp) paired-end reads were obtained using Illumina MiSeq for the proband’s and mother’s microdissected probes and were aligned to the latest build of human reference genome assembly hg38/GRCh38. The DOPseq analyzer pipeline was employed for target region identification [3]. Raw reads were trimmed to remove sequencing adapters and amplification primers: 45,340 (16,780,431 bp, 68.6% of the initial length) and 43,989 (16,563,198 bp, 65.7% of the initial length) reads remained, respectively. After filtering by mapping quality and alignment length, 7349 and 7961 high-quality unique alignments, respectively, were left. Libraries of microdissected chromosomes represent a pool of sequences from a target chromosome passed through the step of whole-genome amplification and low-coverage sequencing. Stringent quality controls are necessary to remove low-grade contamination with sequences of the human operators, sequencing errors, and artifacts caused by low sequencing coverage, thus resulting in a small number of high-quality alignments of target sequences. Alignments were merged into positions (3404 and 3767, respectively) with a total length of 344,487 and 417,964 bp and the average coverage of 2.16 and 2.11 (Additional file 1: Figure S1). After manual verification, two breakpoint regions were detected: HSA9:25,928,021-26,157,441 (9p21.2 band) and HSA15:30,552,104-30,765,905 (15q13.2 band) (build 38). These breakpoint regions’ coordinates were uploaded to the UCSC Genome Browser for breakpoint analysis. Using available tracks, enrichment with repeated elements and segment duplications were checked. Genetic content of the breakpoint regions was analysed (Additional file 1: Table S2). The breakpoint area on HSA15 was located in the region with a large segmental duplication (Fig. 2).

### Brain MRI of the carriers of chromosomal rearrangements

#### Clinical evaluation

**Fig. 3:**
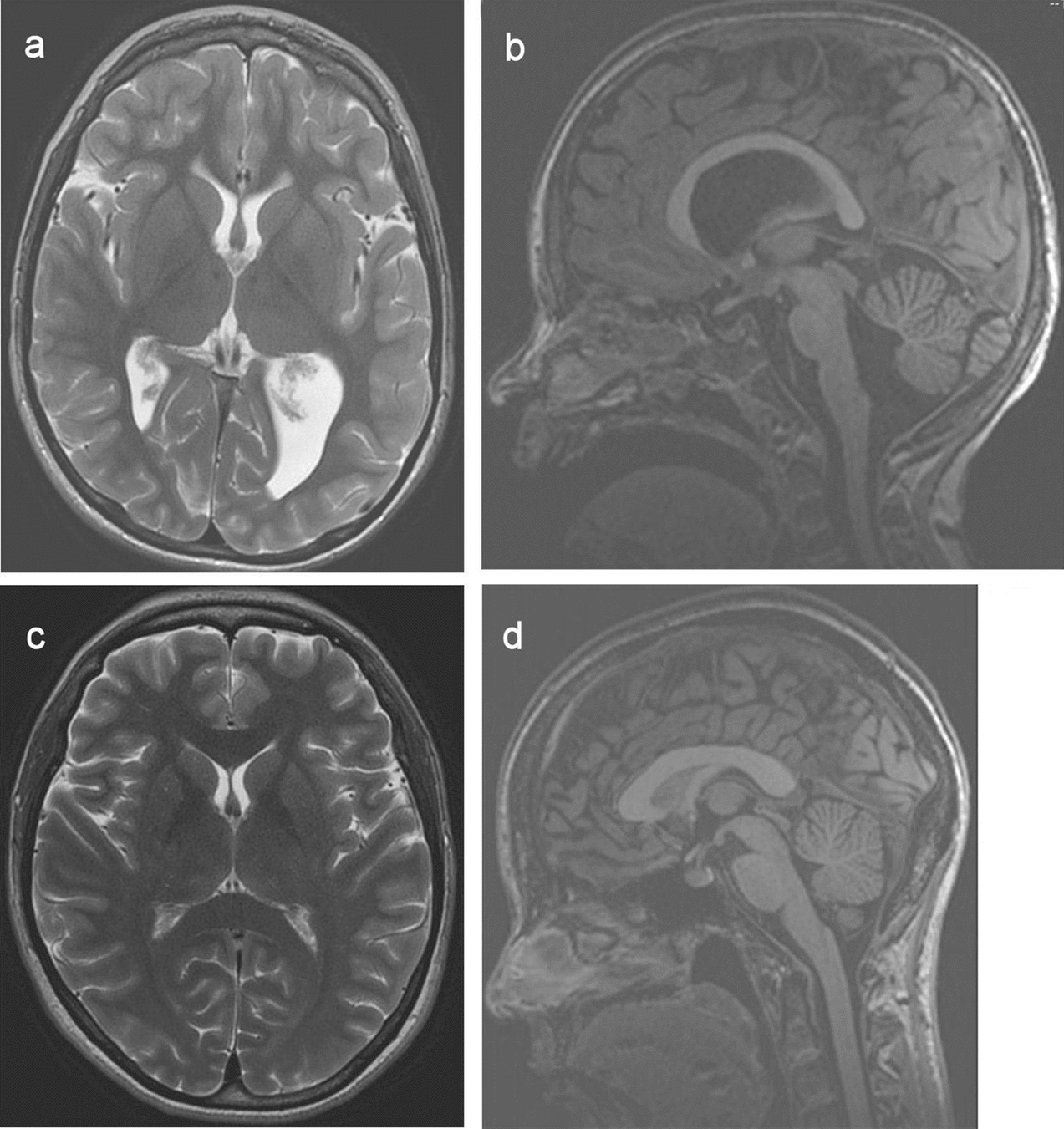
**a** MRI in the axial plane, T2–WI (proband). **b** MRI in the sagittal plane, T1–WI (proband). There is moderate thinning of the corpus callosum, reduced size of the pons, and aplasia of lower segments of the cerebellar vermis (Dandy–Walker variant); chiasm, quadrigeminal, interpeduncular, more closely tank expanded. **c** MRI in the axial plane, T2-WI (mother); no pathology detected. **d** MRI in the sagittal plane, T1–WI (mother). No pathology detected

Routine clinical examination of the child’s brain MRI data revealed several structural (developmental) anomalies, such as corpus callosum dysgenesis and caudal segments of cerebellar vermis hypo/aplasia (Dandy–Walker variant) (Fig. 3).

#### Automatic MR-morphometry

According to FreeSurfer basic segmentation analysis, the child showed a severe decrease in total subcortical gray matter volume, both thalami, putamen and pallidum volumes, hippocampi, and in the brainstem. In addition, an increase in total ventricular volume was detected (compensatory hydrocephalus). The results are shown in Additional file 1: Table S3. No significant deviations of subcortical volumes and ventricle volumes from the normative values were found in the mother.

**Fig. 4:**
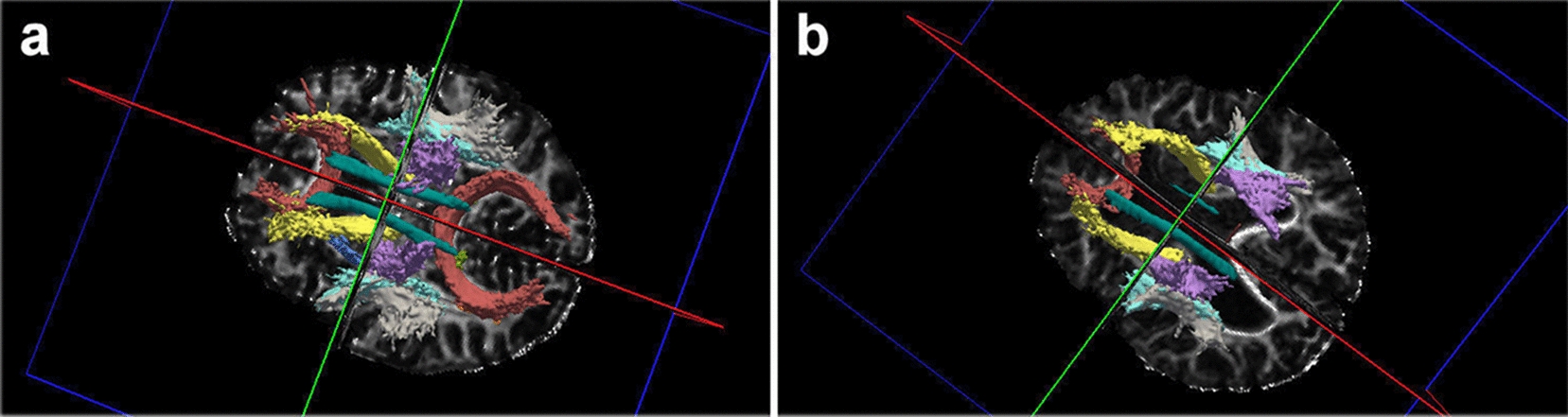
Results of the TRAKULA analysis: **a** The mother’s structural connectivity profile: all of the main white matter tracts (cingulate, CST, arcuate, and forceps major and minor) are seen, with normal length and thickness, symmetrically. **b** The child’s structural connectivity profile: there is marked thinning of all white matter tracts, mainly of the left cingulate (highlighted in dark green) and forceps major (red)

We noted no significant deviations of the mother’s DTI parameters of diffusivity and structural connectivity from the data obtained on healthy volunteers. An analysis of the child’s DTI revealed structural connectivity lesions, mainly in the occipital lobes: a pronounced decrease in tracts’ volumes and in the fractional anisotropy coefficient from forceps major. In addition, there was a diffuse decrease in the fractional anisotropy coefficient in the regions of white and gray matter in the child compared to his mother (Additional file 1: Table S4). Analyses of the FA coefficient of different parts of the corpus callosum indicated that the largest changes affected its posterior sections, while the anterior and central regions were virtually unaffected by the pathological process. The TRAKULA analysis revealed that structural brain connectivity is broken mainly in posterior brain regions. Figure 4 illustrates the FreeSurfer tractography analysis.

#### fMRI connectivity

The mother’s individual connectivity profile did not differ from that of other healthy volunteers: the main nodes of DMN were visualized, and their connectivity with each other and other functional regions of the cortex were within a reference range.

The child’s fMRI revealed several significant deviations in the individual functional connectivity profile.

### The posterior cingulate cortex (PCC; Fig. 5a) and the medial prefrontal cortex (MPFC; Fig. 5b)

**Fig. 5:**
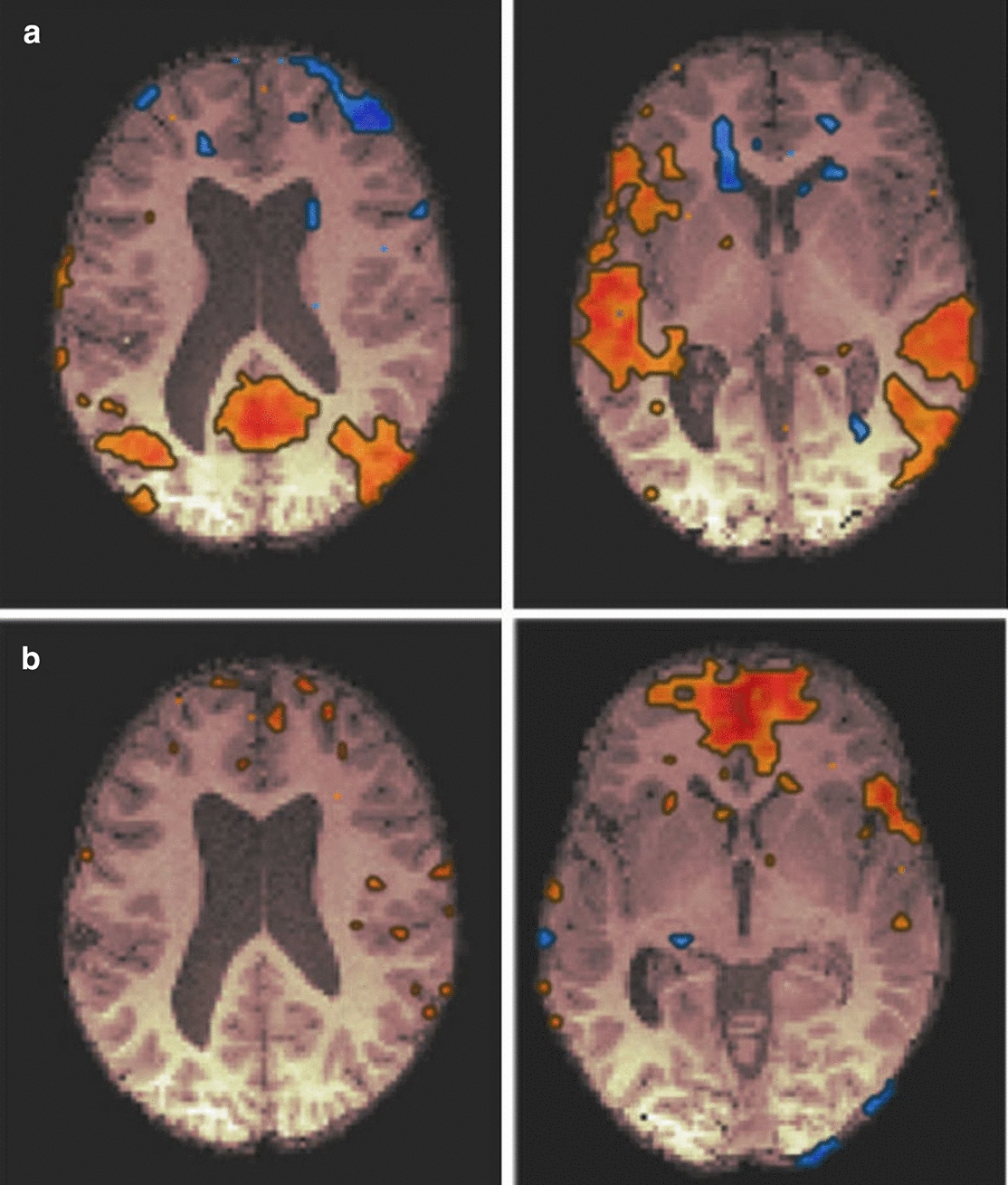
**a** The PCC functional connectivity profile. On the left: the z = 18 level, on the right: the z = 0 level. The absence of connectivity between the PCC and MPFC (the anterior DMN hub) and increased connectivity between the PCC and middle temporal gyri cortex is shown. **b** The MPFC functional connectivity profile. On the left: the z = − 6 level, on the right: the z = 16 level. There is increased neuronal activation within the MPFC and decreased connectivity between the MPFC and other functional brain regions

The correlation coefficient within the PCC was ~ 0.7; an increased correlation with lower parietal lobules (PL, R & L) was detected: ~ 0.7 (for the mother: ~ 0.5); no correlation with the activity of the medial prefrontal cortex (MPFC) was found (complete separation of the anterior and posterior DMN nodes); in addition, increased connectivity between the PCC and middle temporal gyri (right and left), was revealed (~ 0.6); a negative correlation of the PCC with nodes of other functional networks was not detected (a sign of DMN isolation).

The correlation coefficient within the MPFC was ~ 0.7 (with somewhat increased area of activation and neighboring regions); no correlations with inferior PLs (R & L) and PCC were found; a decreased correlation of MPFC with nodes of other functional networks was detected.

### Inferior parietal lobules (PL, R & L)

The PL connectivity profile was very similar to the PCC connectivity profile, and a correlation of PL activity with MPFC activity was not detected (a sign of separation of DMN nodes).

## Discussion

One of the rarest subgroups of sSMC is the "complex" marker chromosomes. A "complex" is an sSMC that is composed of material derived from more than one chromosome [1, 19]. A little more than 400 of such complex sSMCs (8.4% of all sSMC cases) have been characterized in the literature by now and summarized in the sSMC database (http://cs-tl.de/DB/CA/sSMC/0-Start.html) [20, 21].

In general 70% of sSMC cases are *de novo*, whereas for complex sSMC this value is almost 2 times lower and the remaining 64% are parentally derived as a result of balanced translocation transmission [22]. In most cases, supernumerary marker chromosomes associated with congenital malformations and intellectual disability are characterized by the presence of euchromatin segments, which determine the degree and type of pathology [1]. Therefore, research on the composition of marker chromosomes and determination of exact boundaries of rearrangements is an important task of cytogenetic diagnosis.

The proband with a developmental delay and an unidentified marker chromosome was referred for cytogenetic analysis. By microdissection and whole-chromosome probe sequencing followed by bioinformatic analysis, we determined the breakpoint regions. This is a unique case of rearrangement 47,XY,+der(15)t(9;15)(p21.2;q13.2) with breakpoints regions located at HSA9:25,928,021-26,157,441 and HSA15:30,552,104-30,765,905 inherited from the mother, who is a carrier of balanced reciprocal translocation 46,XX,t(9;15)(p21.2;q13.2), likely resulting from 3:1 malsegregation during maternal gametogenesis.

The breakpoint region on chromosome 9 is located in the gene desert area. The only genetic elements of the region are the long noncoding RNA (lncRNA) AL353753.1 gene with an unknown function and pseudogene FAM71BP1. The breakpoint region on chromosome 9 has several areas of high homology with the breakpoint region on chromosome 15 that may have served as a substrate for the balanced-reciprocal-translocation event.

**Fig. 6:**
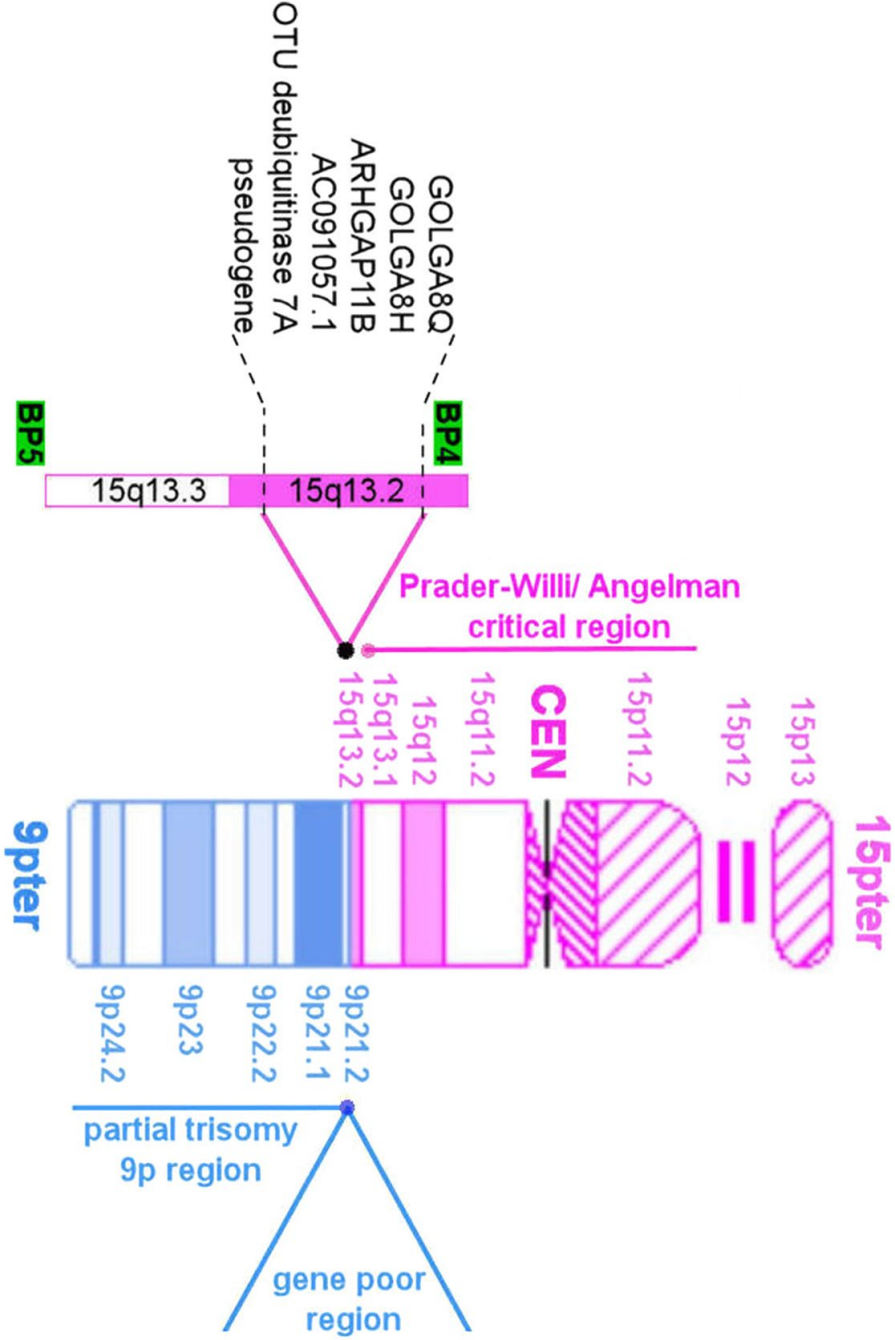
The idiogram of the proband’s supernumerary chromosome

In contrast, the breakpoint region on chromosome 15 is different and rich in genomic elements. The proximal area of 15q is highly unstable because of six low-copy repeat (LCR) elements, in other words, segmental duplications located at each of the six previously described breakpoints (BP1–BP6) [23, 24]. LCRs may underlie a greater proportion of human phenotypic variation and disease than previously recognized [25–28]. Highly homologous LCR structures can act as recombination substrates [29–31]. The discovered breakpoint region of the proband is located in the BP4–BP5 area (Fig. 6). The area studied here includes large segmental duplications caused by the primate-specific chromosome 15 palindromic GOLGA8 repeat (Figs. 2, 6). These areas of segmental duplications arose recently, when ancestral *Homo sapiens* was diverging from archaic hominins [32]. Palindromic architecture of the GOLGA8 core duplicon causes both evolutionary and disease-related instability of chromosome 15. According to one hypothesis [32], the presence of palindromic structures might have contributed to a failure of the replication fork. Due to the homology of GOLGA repeats, recombination might have occurred in a nonallelic way creating an opportunity for the chromosomal breaks. The described case is consistent with the literature data and allows us to assume that the rearrangement in the BP4–BP5 area is the hotspot where the mother has a gap on chromosome 15.

There are many reports on rearrangements occurring separately in the regions of chromosomes 9 and 15 affected in the case under study. The ChromosOmics database details a number of cases with partial trisomy of several chromosomes translocated to der(9) (chromosomes 1, 3, 6, 7, 10, 16) or der(15) (8, 10, 13, 16, 17, 18, 21, 22, Y) [33, 34]. Few cases have been reported on complex sSMC involving the elements of chromosomes 9 and 15 with limited information on the phenotypic features of carriers [20, 35–38]. The proband’s supernumerary chromosome has an additional region of maternal 15pter-q13.2 comprising one extra copy of the Prader–Willi/Angelman critical region (PWACR). Therefore, the patient may have some phenotypic signs characteristic of patients with the Dup(15q) including maternal interstitial 15q11.2-q13.1 duplication or isodicentric chromosome 15. The change in the number of maternal copies of the PWACR may have affected the functioning of the imprinting center and have led to neurodevelopmental problems in the probands [26, 27].These abnormalities manifest themselves as a global developmental delay, intellectual disability, autism spectrum disorders, or epilepsy [39]. Despite the presence of specific electroencephalogram variant (beta EEG) waves of Dup(15q) syndrome [40–43], MRI does not yield abnormal findings in most cases [40, 44, 45] or shows nonspecific changes such as an increase in pericerebral spaces and thinning of the corpus callosum [42]. More recent pathological reports indicate high prevalence of heterotopia and dysplasias in the hippocampus [39, 46].

The second segment of the marker is a part of chromosome 9p material. It is known that trisomy 9p is the fourth most frequent chromosome anomaly [47]. Nonetheless, now there is no consensus on the critical region leading to the 9p trisomy phenotype–genotype correlation [48–53]. Direct duplication of 9p is a very rare event [54]. As a rule, trisomy 9p is caused by a parental translocation between chromosome 9 and another autosome [55]. Since the original publication by Rethore et al. [56], trisomy 9p has become a clinically recognizable syndrome [57]. Intellectual disability is an almost ever-present feature [58] (there are exceptions [59]). An MRI in a patient with partial trisomy 9p often displays Dandy–Walker malformation, which is characterized by a hypoplastic inferior cerebellar vermis and hypoplastic cerebellar peduncles [60] as well as ventriculomegaly [60–62].

Identification of genotype–phenotype correlations in the proband at the brain level is complicated by the presence of contaminant partial trisomy of the other chromosome in complex sSMC. Thus, dysgenesis of the corpus callosum could have been induced either by a duplication of the PWACR or by partial 9p trisomy. After analyzing the literature [39, 42, 46, 57, 62–65] and our data (Table 1), we can hypothesize that the abnormality involving the amygdala hippocampus complex is due to trisomic 15pter-q13.2. We suppose that a greater number of 9pter-9p21.2 maternal copies is a risk factor for hypo/aplasia of caudal segments of the cerebellar vermis (Dandy–Walker variant) and enlargement of the posterior fossa. There is not enough evidence to link the decrease in subcortical gray matter volume and brainstem volume to either 15pter-q13.2 or 9pter-9p21.2. Nonetheless, changes are detectable in both grey and white matter in case of PWACR deletion or maternal unisomy of chromosome 15 in patients with Prader–Willi syndrome [66, 67]. The patient has several phenotypic features that are not consistent with either syndrome. The traits either may have developed through an interaction of the two karyotypic abnormalities or may reflect the amount of additional genetic material present in his genome. Among few reported cases of derivative chromosome resulting from the translocation of 9 and 15, the case of der(15)t(9;15)(p11;q11) has detailed phenotypic and MRI description [37]. A number of features in three patients intersects with the proband here: developmental and language delays, low set ears, strabismus, and signs of hydrocephalus.

**Table 1:**
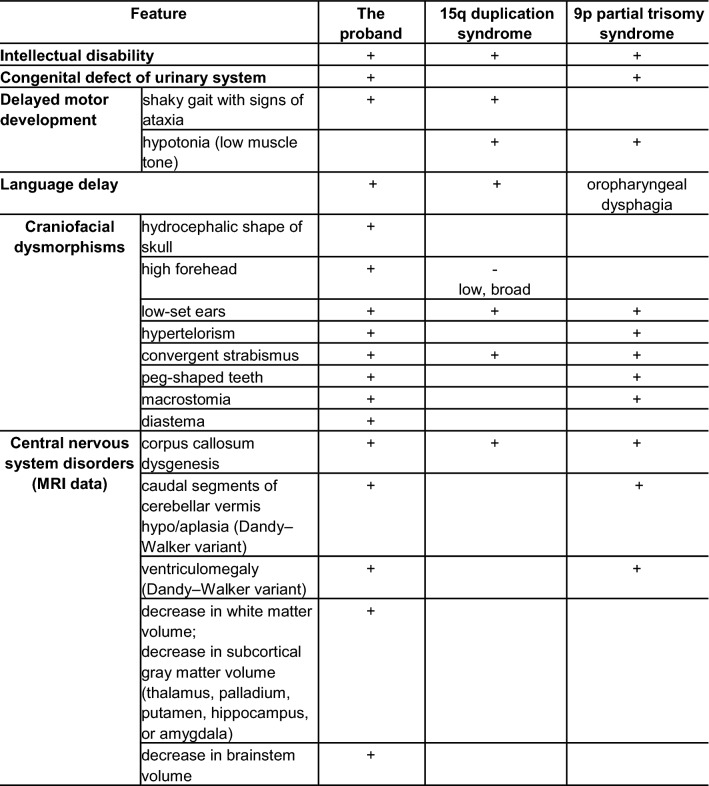
Phenotypic features of the proband in comparison to Dup(15q) and 9p partial trisomy syndromes

(+) Present and described in the literature at least once; (−) absent; ( ) not mentioned in reports [38–40, 43, 44, 50, 54, 60, 67–74]

The two identified breakpoint regions carrying uncharacterized transcripts, pseudogenes, and repeats do not contain any important genes and have no effect on the mother’s phenotype. We suppose that repeated elements inside the found breakpoint regions might have contributed to the rearrangement. We noted both unique features and a high correlation with two syndromes of trisomy on 9p and 15. We recommend MRI for all patients with a developmental delay, especially in cases with identified rearrangements, to accumulate more information on brain phenotypes related to chromosomal syndromes because these data may help to identify the causative chromosomal regions.

## Supplementary Information

**Additional file 1**.

## Data Availability

The data and material used or analysed during the current study are available from the corresponding author on reasonable request.

## Abbreviations

MRI: Magnetic resonance imaging
fMRI: Resting-state functional MRI
sSMCs: Supernumerary marker chromosomes
Mbp: Million base pairs
FISH: Fluorescence in situ hybridization
PWACR: Prader–Willi/Angelman critical region
NGS: Next-generation sequencing
MAPQ: Mapping quality
DTI: Diffusion tensor imaging
PCC: Posterior cingulate cortex
MPFC: Medial prefrontal cortex
