# Supplementary material for: A rare familial rearrangement of chromosomes 9 and 15 associated with intellectual disability: a clinical and molecular study
Source: Mol Cytogenet. 2021 Oct 4;14:47. doi: 10.1186/s13039-021-00565-y (PMC8489072; doi:10.1186/s13039-021-00565-y)
Supplement: Supplementary file 1 — Additional file 1. [file 13039_2021_565_MOESM1_ESM.docx]

**Supplemental files**

**
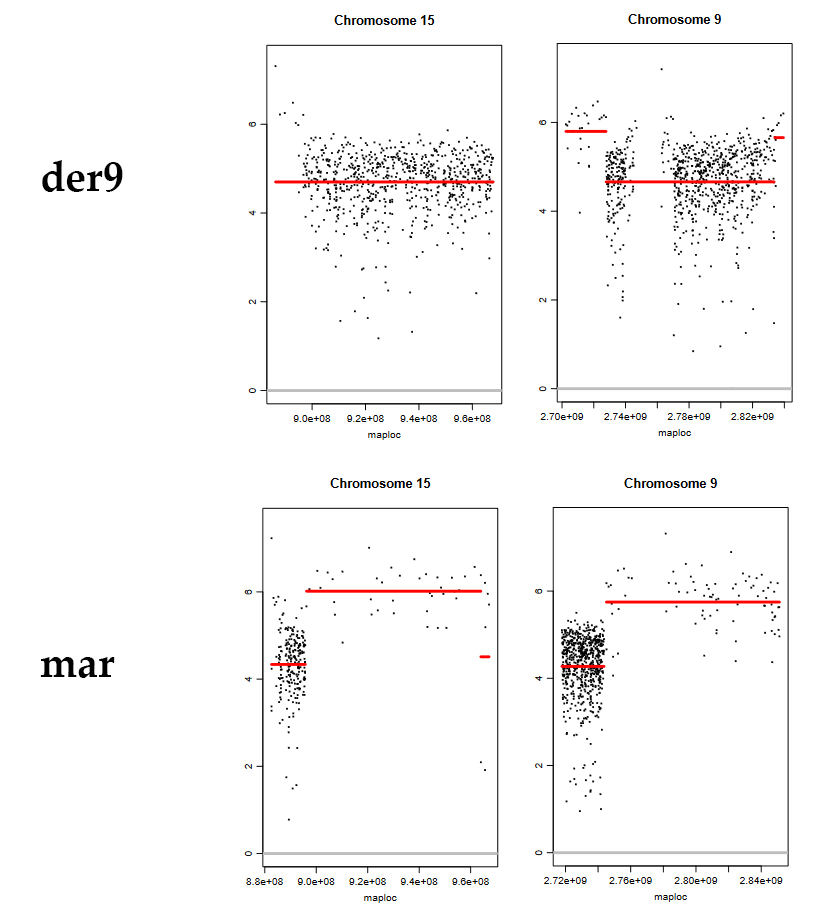
**

**Fig. S1** Rainfall plots of distances between mapped read positions. Only chromosomes 15 and 9 are presented. The red lines are mean values inferred by the DOPseq analyzer pipeline, and the black dots correspond to distances between mapped positions covered by reads on a log scale.

**Supplemental Table 1** Names and MNI coordinates of ROIs.

| Functional connectivity network | ROI (DMN node) | MNI coordinates of the center |
| --- | --- | --- |
| DMN | posterior cingulate cortex (PCC) | 1; -61; 38 |
|  | medial prefrontal cortex (MPFC) | 1; 55; -3 |
|  | inferior parietal lobule (PL, R & L) | 47; -67; 29 (R)  -37; -67; 29 (L) |

**Supplemental Table 2** Genetic content of the identified breakpoint regions on chromosomes 9 and 15.

| **Gene name** | **Gene type** | **Start** | **End** |
| --- | --- | --- | --- |
| **Chromosome 9** | | | |
| Breakpoint region of HSA9 |  | 25,928,021 | - |
| *FAM71BP1* | processed pseudogene | 25,937,912 | 25,938,434 |
| *AL353753.1* | lncRNA | 26,066,675 | 26,118,408 |
| Breakpoint region of HSA9 |  | - | 26,157,441 |
| **Chromosome 15** | | | |
| Breakpoint region of HSA15 |  | 30,552,104 | - |
| *GOLGA8Q* | protein coding | 30,552,105 | 30,562,501 |
| *RN7SL796P* | miscellaneous RNA | 30,560,644 | 30,560,934 |
| *DNM1P50* | unprocessed pseudogene | 30,567,822 | 30,571,089 |
| *ULK4P2* | transcribed unprocessed pseudogene | 30,572,738 | 30,600,647 |
| *AC026150.1* | small nucleolar RNA | 30,581,184 | 30,581,316 |
| *GOLGA8H* | protein coding | 30,604,126 | 30,614,561 |
| *AC026150.4* | lncRNA | 30,607,695 | 30,608,193 |
| *RN7SL628P* | miscellaneous RNA | 30,612,724 | 30,612,960 |
| *AC091057.4* | lncRNA | 30,616,958 | 30,617,749 |
| *AC091057.3* | lncRNA | 30,616,998 | 30,625,773 |
| *ARHGAP11B* | protein coding | 30,624,494 | 30,649,529 |
| *AC091057.6* | protein coding | 30,624,548 | 30,685,606 |
| *AC091057.7* | transcribed unprocessed pseudogene | 30,644,633 | 30,723,495 |
| *AC091057.1* | lncRNA | 30,658,717 | 30,765,905 |
| *AC091057.2* | miscellaneous RNA | 30,673,750 | 30,673,843 |
| *AC091057.5* | transcribed unprocessed pseudogene | 30,750,406 | 30,759,859 |
| Breakpoint region of HSA15 |  | - | 30,765,905 |

**Supplemental Table 3** Volumes of subcortical structures in the patient in comparison with standard values.

| Structure | Observed V, μl | Predicted V, μl | PI 95% | p |
| --- | --- | --- | --- | --- |
| Brainstem | 13151 ** | 18186 | 14382-21791 | 0.005 |
| Thalamus R | 5175 ** | 7134 | 5968-8299 | 0.009 |
| Thalamus L | 6227 * | 7830 | 6294-9366 | 0.02 |
| Caudate R | 4467 | 4225 | 3297-5153 | 0.305 |
| Caudate L | 4198 | 3998 | 3144-4853 | 0.323 |
| Pallidum R | 1359 ** | 1944 | 1542-2345 | 0.002 |
| Pallidum L | 1091 *** | 1880 | 1413-2347 | 0.000 |
| Putamen R | 4050 *** | 6734 | 5522-7947 | 0.000 |
| Putamen L | 2825 *** | 6457 | 5145-7769 | 0.000 |
| Hippocampus R | 3318 ** | 4353 | 3594-5113 | 0.004 |
| Hippocampus L | 3529 * | 4336 | 3568-5104 | 0.02 |
| Amygdala R | 416 *** | 810 | 581-1038 | 0.000 |
| Amygdala L | 414 ** | 799 | 544-1054 | 0.002 |
| Total subcortical gray matter volume | 47377 *** | 64804 | 58039-71570 | 0.000 |
| Total ventricular volume | 76608 *** | 12614 | 6112-26032 | 0.000 |

**Supplemental Table 4** A coefficients of the child’s (compared to mother’s) intracranial structures.

| **Structure** | **FA (child)** | **FA (mother)** |
| --- | --- | --- |
| Corpus callosum (rostral part) | 0.64 | 0.62 |
| Corpus callosum (anterior body) | 0.54 | 0.44 |
| Corpus callosum (central body) | 0.5 | 0.53 |
| Corpus callosum (posterior body) | 0.45 | 0.65 |
| Corpus callosum (splenium) | 0.57 | 0.79 |
| Thalamus R | 0.28 | 0.45 |
| Thalamus L | 0.29 | 0.41 |
| Hippocampus R | 0.13 | 0.17 |
| Hippocampus L | 0.14 | 0.16 |
| Hemispheric white matter | 0.2 | 0.3 |
